# Synthesis, Characterization, and Adsorptive Properties of Fe_3_O_4_/GO Nanocomposites for Antimony Removal

**DOI:** 10.1155/2017/3012364

**Published:** 2017-07-20

**Authors:** Xiuzhen Yang, Tengzhi Zhou, Bozhi Ren, Zhou Shi, Andrew Hursthouse

**Affiliations:** ^1^College of Civil Engineering, Hunan University of Science and Technology, Xiangtan 411201, China; ^2^College of Civil Engineering, Hunan University, Changsha 410082, China; ^3^Key Laboratory of Building Safety and Energy Efficiency, Ministry of Education, Hunan University, Changsha 410082, China; ^4^School of Science & Sport, University of the West of Scotland, Paisley PA1 2BE, UK

## Abstract

A magnetic Fe_3_O_4_/GO composite with potential for rapid solid-liquid separation through a magnetic field was synthesized using GO (graphene oxide) and Fe_3_O_4_ (ferriferous oxide). Characterization of Fe_3_O_4_/GO used scanning electron microscope (SEM), X-ray diffractometer (XRD), Fourier transform infrared spectrometer (FT-IR), and Vibrating Sample Magnetometer (VSM). A number of factors such as pH and coexisting ions on adsorbent dose were tested in a series of batch experiments. The results showed that GO and Fe_3_O_4_ are strongly integrated. For pH values in the range of 3.0~9.0, the removal efficiency of Sb(III) using the synthesized Fe_3_O_4_/GO remained high (95%). The adsorption showed good fit to a pseudo-second-order and Langmiur model, with the maximum adsorption capacity of 9.59 mg/g maintained across pH 3.0–9.0. Thermodynamic parameters revealed that the adsorption process was spontaneous and endothermic. Analysis by X-ray photoelectron spectroscopy (XPS) showed that the adsorption process is accompanied by a redox reaction.

## 1. Introduction

Antimony has recently gained considerable attention as a toxic heavy metal [1]. By binding with sulfhydryl groups inside human body, antimony and antimony compounds can interfere in enzyme activity or destroy intracellular ionic equilibrium which leads to cell hypoxia, causing metabolic disorders and injury to the nervous system and other organs [2].

Antimony has been classified as a priority pollutant by European Union (EU) and United States Environmental Protection Agency (EPA) in 1976 and 1979, respectively [3, 4]. The Environmental Protection Department of Japan also listed it as a pollutant of concern and stipulated a maximum acceptable concentration of 2 ug/L [5]. In China, the maximum concentration of antimony limited by GHZBl and Drinking Water Health Standards is 5 ug/L [6], which is consistent with the standards of World Health Organization [7].

China has the largest reserve and capacity for production of antimony in the world [8]. Over 80% of total world antimony production is in China in the past several decades, and use is wide spread as a catalyst in cPET production, in flame retardants, in alloys, and in the electronics industry. The main production areas are in Hunan in the southwest of China, where the 100-year-old mine, Xikuangshan, is known as the World Antimony Capital. The region has a history of nearly 200 years of antimony ore production and, therefore, antimony pollution in this region is of concern. Research shows that the antimony content of water in the mining area can exceed 7,000 ug/L [9]. The severity of antimony pollution has threatened the health of residents surrounding the mine. Therefore, cost effective methods to control antimony pollution in China, especially in the southwest, have become an imperative.

Toxicity of antimony is mainly affected by its valence state and the nature of compounds with the toxicity of trivalent antimony being ten times higher than that of pentavalent antimony [10–12]. Hence, trivalent antimony is chosen as the target oxidation state of this pollutant.

A variety of methods have been developed to remove antimony from solution, of which approaches using adsorption are popular due to the effectiveness and availability of of reactive solid phases. Due to the huge specific surface area (2,630 m^2^/g) [13], Graphene Oxide (GO) has been widely applied in water treatment; however, its strong sorption makes it difficult to desorb after reaction. The preparation of Fe_3_O_4_/GO nano composites for effective pollutant removal has been demonstrated [14] showing strong affinity and reversibility for a number of pollutants and was prepared in this experiment to evaluate removal of antimony from water.

## 2. Materials and Methods

### 2.1. Reagent and Instrument

*Raw Material and Reagent*. Graphene Oxide (prepared by modified Hummers' method) and the regents (antimony potassium tartrate, sodium hydroxide, hydrochloric acid, etc.) were analytically pure.

*Key Instruments*. The key instruments are Scanning electron microscope (JSM-6700F, Japan, JEOL); X-ray diffractometer (D8/ADVANCE, Germany, BRUKER); Fourier transform infrared spectrometer (IRAffinity-1, Japan Shimadzu); magnetometer (MPMS-XL-7, the United States, Quantum Design Company); atomic absorption spectrophotometer (AA-7000, Japan, JEOL).

### 2.2. Preparation of Fe_3_O_4_/GO

Fe_3_O_4_ particles were prepared by coprecipitation method. The successful in situ growth of Fe_3_O_4_ nanoparticles on GO surface during the synthetic process of Fe_3_O_4_/GO composites was ascribed to the oxygen-containing functional groups of GO. The as-synthesized composites in this study not only have the excellent adsorption properties of GO, but also possess the superparamagnetism of Fe_3_O_4_ nanoparticles. Hence, the proportion of Fe_3_O_4_ to GO needed optimization during the preparation of composites to take advantage of the strong adsorption properties of GO and the magnetism of Fe_3_O_4_.

GO (15 mg) was dispersed into DI water (30 ml) by ultrasonication for 30 min. To this suspension, 50 ml solution of FeCl_3_ (110 mg) and FeCl_2_ (43 mg) in DI water was added at room temperature. Then the temperature was raised to 85°C and a 30% ammonia solution was added increasing the pH to 10.0. After being rapidly stirred for 1 h the solution was cooled to room temperature. The resulting black precipitate was centrifuged at 4500 rpm for 10 min and washed three times with DI water and finally dried in a vacuum oven at 60°C for overnight to yield the Fe_3_O_4_/GO composite.

### 2.3. Adsorption Experiments

The model wastewater with varying concentration of Sb(III) was prepared using antimony potassium tartrate. Aliquots of 50 ml of model wastewater with the appropriate Sb concentration and a quantity of Fe_3_O_4_/GO were added. Batch adsorption experiments were undertaken at a constant temperature with shaking prior to magnetic separation of the residue. The supernatant was filtered using a 0.45 um membrane, and the concentration of residual Sb(III) in solution was measured by atomic absorption spectrophotometry. The factors influencing sorption investigated included the initial pH value, the Fe_3_O_4_/GO dose, adsorption time, and temperature.

The removal rate (%) was calculated from

(1) $$\begin{array}{c} \text{Removal rate}=\frac{c_{0}-c_{t}}{c_{0}}\times 100\%, \end{array}$$

where *c*_0_ and *c*_*t*_ are the initial and final concentrations of Sb(III) in the solution (mg/l), respectively.

**Figure 1:**
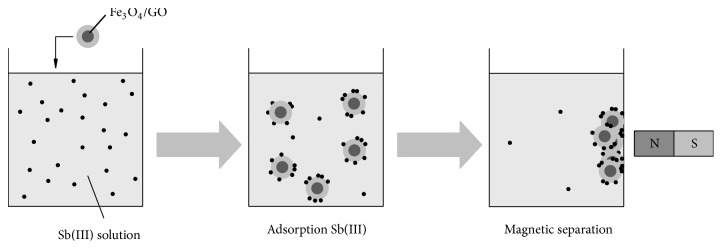
The adsorption experiment process.

It was a static adsorption experiment, and the experimental arrangement is shown in Figure 1.

## 3. Result and Discussion

### 3.1. Solid Characterization

#### 3.1.1. SEM

**Figure 2:**
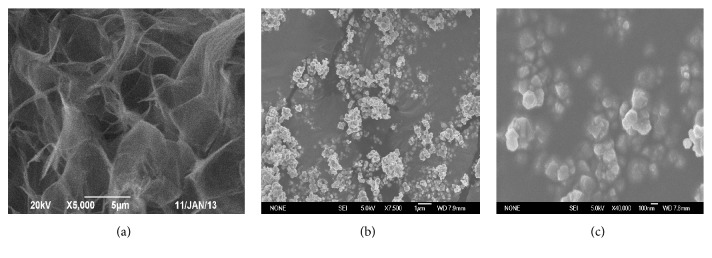
(a) SEM images of GO; (b) and (c) SEM images of Fe_3_O_4_/GO at different magnification.

Figure 2 showed the SEM images of GO and Fe_3_O_4_/GO at different magnification. The SEM images (b) and (c) show the Fe_3_O_4_ Nps added as bright dots that were uniformly spread over the surface of the GO, which indicated that the Fe_3_O_4_ was coated on the GO successfully. Magnetic Fe_3_O_4_ nanoparticles formed in alkaline condition could be firmly loaded on GO because the Fe^3+^ and Fe^2+^ in solution reacted with the carboxyl functional groups of GO to form coordination compounds that were easily deposited on the surface of GO.

The reaction processes are previously identified as (2)–(4) [15].

(2) 2Fe2++GO⟶2Fe3++rGO

(3) Fe2++2Fe3++8OH−⟶Fe3O4+4H2O

(4) 3Fe2++GO8OH−⟶Fe3O4+4H2O+rGO

#### 3.1.2. FT-IR

**Figure 3:**
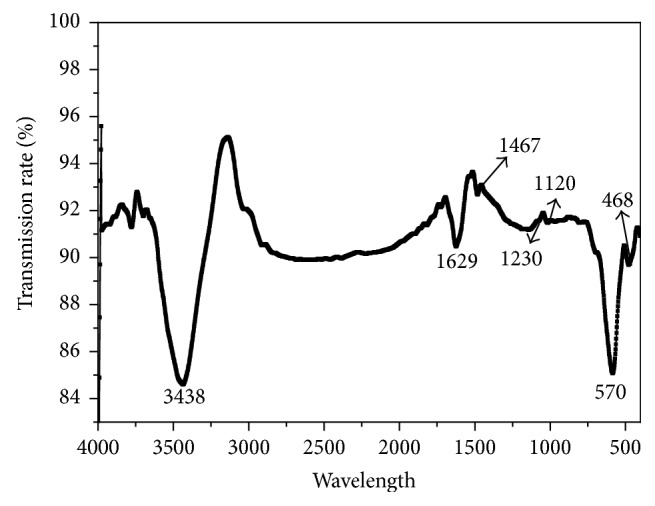
FT-IR patterns of Fe_3_O_4_/GO.

The images in Figure 3 show the FT-IR spectrum for Fe_3_O_4_/GO composites. Two strong vibration stretch peaks appeared at 570 cm^−1^ and 468 cm^−1^, which were characteristic absorption peaks of Fe-O bond [16]. The absorption peaks at 3438 cm^−1^, 1629 cm^−1^, 1230 cm^−1^, and 1120 cm^−1^ were related to the vibration peak of molecular adsorption, the vibration absorption peak of C=O in carboxyl, C=O-H or vibration of ketone skeleton, and the vibration of C-O-C, respectively [17]. The results indicated that a large amount of oxygen-containing functional groups exist on the surface of the GO and Fe_3_O_4_ nanocomposite.

#### 3.1.3. XRD

**Figure 4:**
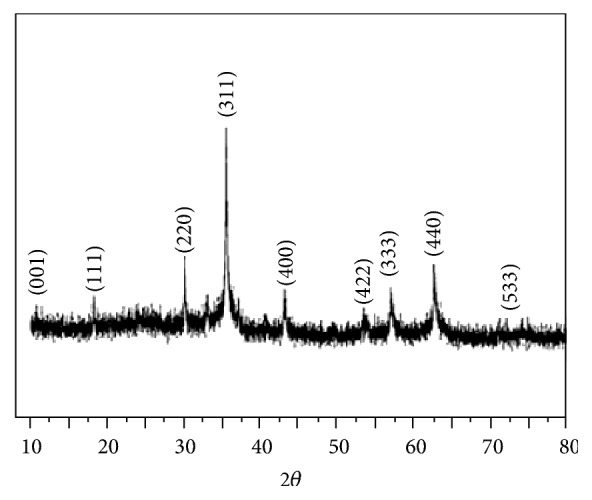
XRD patterns of Fe_3_O_4_/GO.

The powder X-ray diffraction pattern in Figure 4 for Fe_3_O_4_/GO composites shows the diffraction basal spacing (001) is due to the crystalline GO (with 2*θ* at 10.9°). Seven distinct diffraction peaks of varying FWHM are identified for 2*θ* at 30.1°, 35.4°, 43.1°, 53.2°, 62.7°, and 74.0° and correspond to cubic phase crystal structure of Fe_3_O_4_ which were (220), (311), (400), (422), (440), and (533), respectively [18]. Moreover, it can be noted that there was no other impurity peak in XRD, which means the in situ growth of GO and Fe_3_O_4_ did not affect the structure of Fe_3_O_4_ during the synthesis of the Fe_3_O_4_/GO composites. The cubic structure of Fe_3_O_4_ is confirmed.

#### 3.1.4. Vibrating Sample Magnetometer

**Figure 5:**
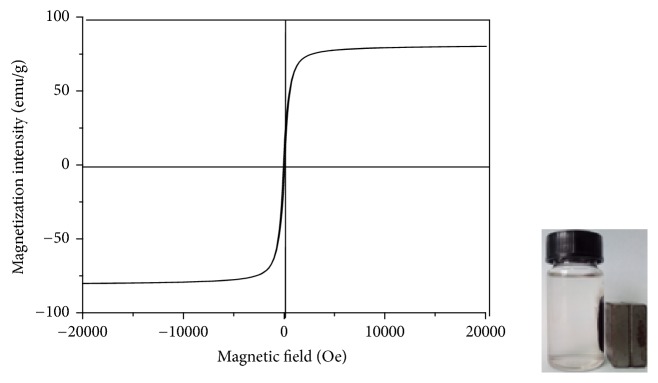
The hysteresis loop of Fe_3_O_4_/GO.

The plot in Figure 5 shows the hysteresis loop of the composite adsorbent. The specific magnetism was up to 87.3 emu/g, which indicated that the composite could achieve fast solid-liquid separation under the applied magnetic field.

The hysteresis loop of Fe_3_O_4_/GO was close to “S,” and the surplus magnetic strength and magnetic coercive force were nearly zero and thus it belongs to soft magnetism. It confirms that Fe_3_O_4_/GO composites were superparamagnetic. The results confirm good separation potential as reported elsewhere, and strong potential for recovery and reuse in sorption treatment systems [19].

### 3.2. Evaluation of the Performance of Fe_3_O_4_/GO Composite in the Adsorption of Sb(III)

#### 3.2.1. The Effect of pH

**Figure 6:**
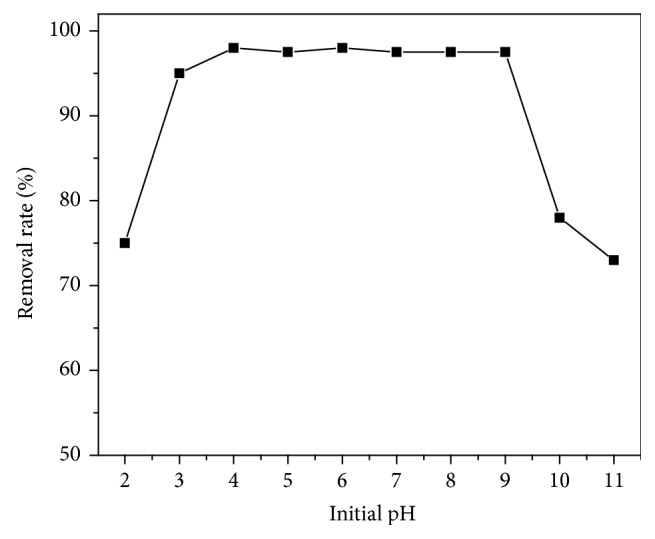
Effects of pH on adsorption efficiency of Sb(III).

An experiment on the effect of pH on Sb(III) adsorption was carried out over the pH range of 2.0–11.0. The initial concentration of antimony was *C*_0_ = 10 mg/L; and the dose of Fe_3_O_4_/GO was 100 mg/50 ml; the reaction temperature was *T* = 298 K; reaction time was *t* = 120 min, with the mixing speed of 120 r/min. Experimental results are shown in Figure 6.

Figure 6 indicated that the adsorption of Sb(III) by using Fe_3_O_4_/GO was not affected in the pH range of 3.0–9.0, and the maximum removal rate was over 95%. This phenomenon is because the species of Sb(III) are pH dependent: when the pH is below 3.0, the predominant species of Sb(III) are SbO^+^ and Sb(OH)^2+^, when the pH ranges from 3.0 to 9.0 Sb(OH)_3_ and HSbO_2_ are the main species of Sb(III), and only SbO^2−^ exists when the pH is above 9.0 [20]. Since the highest adsorption is achieved at pH 3.0–9.0 and pH value of the aquatic environment is usually around 7.0, thus pH 7.0 is selected in further experiments.

#### 3.2.2. Effect of Sorbent Dose

10 × 50 ml samples of Sb(III) solution with an initial concentration of 10 mg/L were taken. The solution was added in a 100 ml polyethylene bottle, and pH was adjusted to 7.0. Finally, the solution was mixed in a constant temperature shaker (298 K) for 120 minutes.

**Figure 7:**
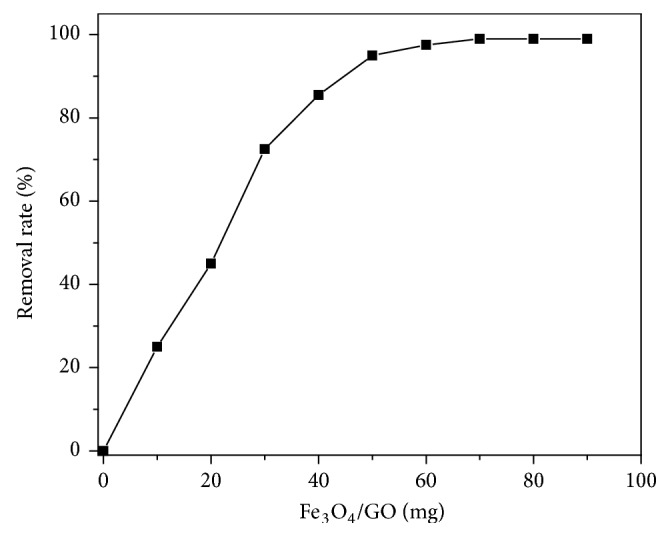
Effects of Fe_3_O_4_/GO dosage on removal efficiency of Sb(III).

As shown in Figure 7, adsorption removal rate of Sb(III) increased with the Fe_3_O_4_/GO dose. Initially, there was a sharp increase in the rate of adsorption, which later slowed down. The adsorption equilibrium was achieved at a dose of 60 mg. This might be explained by the fact that the contact area between adsorbate and adsorbent became larger and the adsorption points of Fe_3_O_4_/GO increased with the addition of Fe_3_O_4_/GO. Furthermore, the difference in Sb(III) concentration between initial adsorbent and adsorbate was relatively large and more Sb(III) could be adsorbed by Fe_3_O_4_/GO, which also leaded to a fast adsorption at the initial stage. With the increase of sorbent dose, adsorption of Sb(III) could reach up to 100%. In subsequent experiments, 60 mg/50 ml was chosen as the optimum dose.

#### 3.2.3. Effect of Coexisting Ions

**Figure 8:**
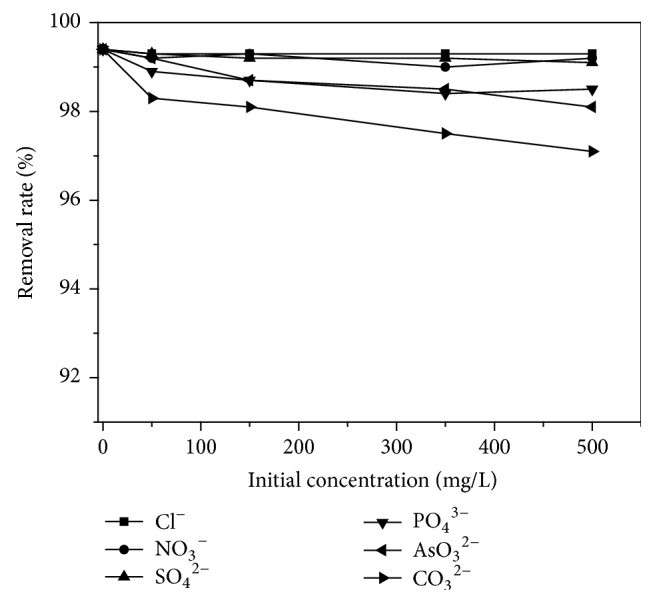
Effects of the coexisting ions.

Effect of various anions on Sb(III) adsorption efficiency was investigated. A series of experiments using Cl^−^, NO_3_^−^, SO_4_^2−^, PO_4_^3−^, AsO_3_^2−^, and CO_3_^2−^ were selected to simulate background interference. Ion concentrations used were 0, 50, 150, 350, and 500 mg/L and the dose was 60 mg/50 ml. The results were illustrated in Figure 8.

As shown in Figure 8, Sb(III) adsorption efficiency was all over 97% for all ions. It should be noted that the removal efficiency showed a relatively low decrease with increase in anion concentration. The order of decrease in removal efficiency was Cl^−^ < NO_3_^−^ < SO_4_^2−^ < PO_4_^3−^ < AsO_3_^2−^ < CO_3_^2−^. Actually, this phenomenon was related to the degree of association of above ions with soluble Sb species impacting on removal efficiency.

### 3.3. Adsorption Kinetics

In order to study the kinetics of Fe_3_O_4_/GO absorbing Sb(III), a series of Sb(III) solutions with concentration of 10 mg/L and initial pH value of 7.0 were taken and 60 mg of Fe_3_O_4_/GO was added to each solution. When temperature was 298 K, the variation of adsorption (*q*_*t*_) with the change of time was measured. Pseudo-first-order kinetic model and pseudo-second-order kinetic model were adapted to analyze characteristics of adsorption kinetics. Model equations are listed as Formula (5) [18].

(5) ln⁡qe−qt=ln⁡qe−k1t,tqt=1k2qe2+1qet,

where *q*_*t*_ and *q*_*e*_ (mg/g) are the amounts of Sb(III) adsorbed onto Fe_3_O_4_/GO at time *t* and at equilibrium, respectively; *k*_1_ is the rate constant, determined by plotting ln⁡(*q*_*e*_ − *q*_*t*_) versus *t*; and *k*_2_ is the rate constant.

**Figure 9:**
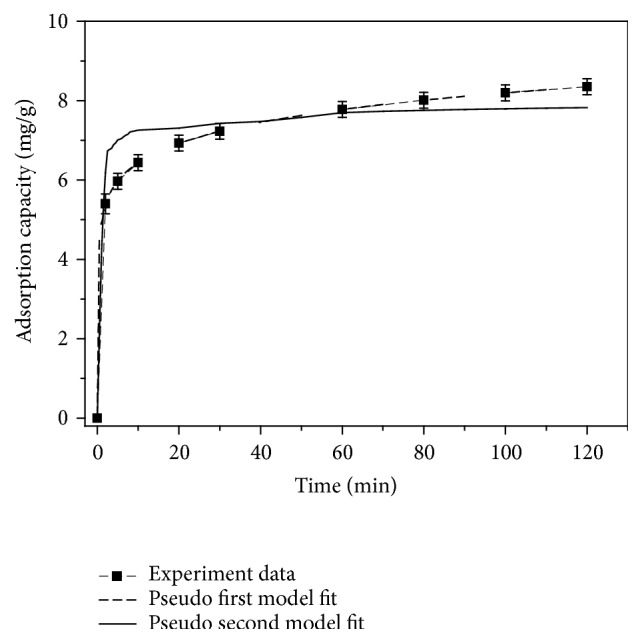
Kinetics models for fitting Sb(III) adsorption using Fe_3_O_4_/GO.

For the first-order kinetic equation of specified initial concentration, ln⁡(*q*_*e*_ − *q*_*t*_) to time (*t*) was plotted, and the linear fitting was done. The first-order and second-order kinetics fit lines (Figure 9) were obtained by the fitted parameters. Similarly, for second-order kinetic equation, *t*/*q*_*t*_ to time was graphed and the linear fitting was undertaken. The second-order kinetics fit line in Figure 9 was based on the best fit parameters.

From Figure 9, it is clear that the adsorption of Sb(III) by Fe_3_O_4_/GO is more suitable for the pseudo-first-order kinetics model which implies a simple sorption mechanism rather than chemical reaction.

### 3.4. Adsorption Thermodynamics

In order to study the thermodynamic characteristics of Sb(III) adsorption by Fe_3_O_4_/GO, a series of Sb(III) solutions with various concentrations and initial pH value of 7.0 were taken. The solutions were put in constant temperature incubation box (298 K) and the adsorption was performed for 24 hours. The equilibrium absorption capacity (*q*_*e*_) as a function of equilibrium concentration (*C*_*e*_) at the different temperatures was plotted as the adsorption isotherms of Sb(III) in Figure 8.

Data were fitted using the Langmuir [21] and Freundlich [22] isotherm models, and the equations for both models are presented as Formula (6).

(6) Ceqe=Ceqmax+1qmaxKL,ln⁡qe=ln⁡KF+1nln⁡Ce,

where *C*_*e*_ is the mass concentration of Sb(III) when the solution is in the state of adsorption equilibrium; *K*_*L*_ is the Langmuir adsorption constant. *K*_*F*_ is a Freundlich constant related to adsorption capacity and 1/*n* is an empirical parameter giving an indication of the favorability of adsorption. It is conducted that when 1/*n* is between 0.1 and 0.5 the adsorbate is easily adsorbed, while when 1/*n* is larger than 2.0 it is difficult to adsorb [6].

**Figure 10:**
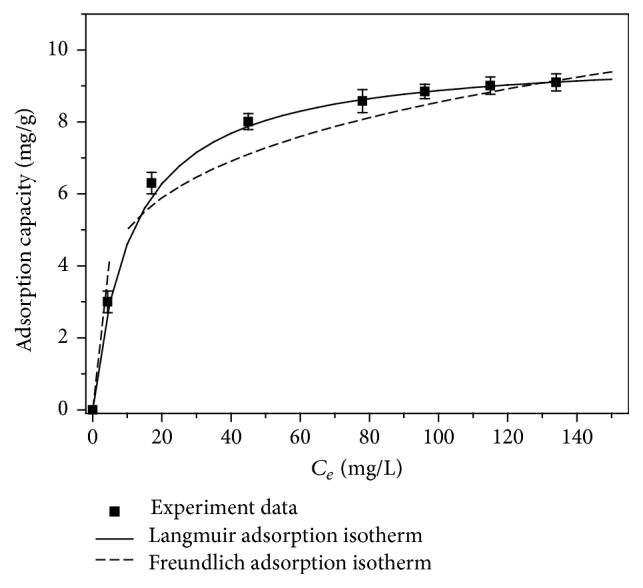
Thermodynamic models for fitting Sb(III) adsorption using Fe_3_O_4_/GO.

From Figure 10, it can be seen that the adsorption reaction matches the Langmuir isothermal adsorption model, which indicated that the adsorption of Sb(III) onto Fe_3_O_4_/GO was the monolayer adsorption. Moreover, adsorbing capacity increased with an increase in temperature. Meanwhile, 0.1 < 1/*n* < 0.5 in Table 1, which meant that the reaction was thermodynamically favourable.

Thermodynamic parameters were calculated by the formulas shown below [23]:

(7) ΔG=−RTln⁡KD,

(8) ΔG=ΔH−TΔS,

(9) ln⁡KD=ΔSR−ΔHRT,

where *K*_*D*_ is the solid-liquid distribution coefficient [24]; Δ*G* is the Gibbs free energy change value in adsorption process, kJ/mol; Δ*H* is the caloric value in adsorption process, kJ/mol; Δ*S* is the entropy change value in adsorption, J/(mol·K).

**Figure 11:**
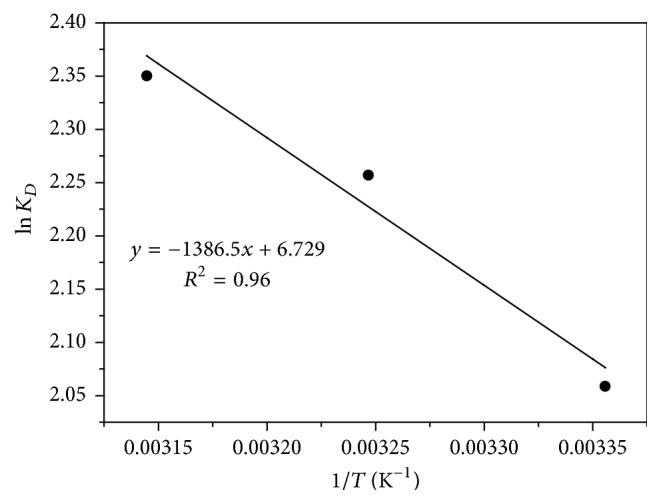
Thermodynamic parameters linear fit.

Based on the experimental results of the adsorption of Sb(III) onto Fe_3_O_4_/GO, the linear imitation was graphed in Figure 11 1/*T* was the abscissa while ln⁡*K*_*D*_ was the ordinate. According to Formula (9) and the obtained slope and intercept of the straight line, Δ*H* and Δ*S* were calculated, and Δ*G* in the corresponding temperature was calculated by Formula (7). The results were presented in Table 2.

Table 2 showed that the adsorption reaction was a spontaneous process when the Gibbs free energy change value (Δ*G*) was below zero. And when Δ*S* > 0, the adsorption process increased the entropy of the process. As for the Δ*H*, the adsorption reaction was an endothermic process when its value was over zero and thus the temperature rise was helpful to move the reaction forward.

### 3.5. Analysis of Adsorption Mechanism

**Figure 12:**
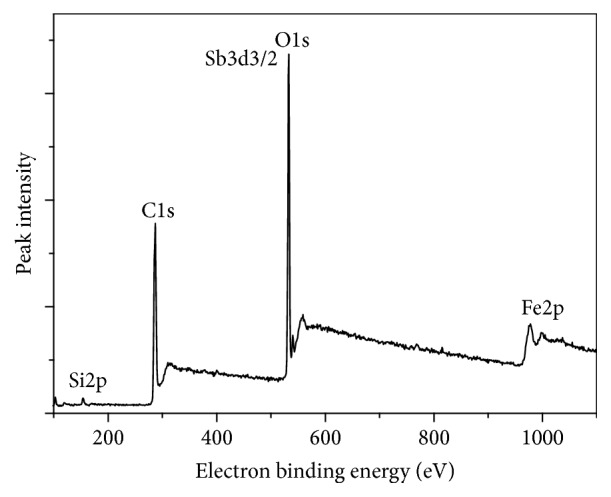
The total element distribution spectrum after adsorption Sb(III).

**Figure 13:**
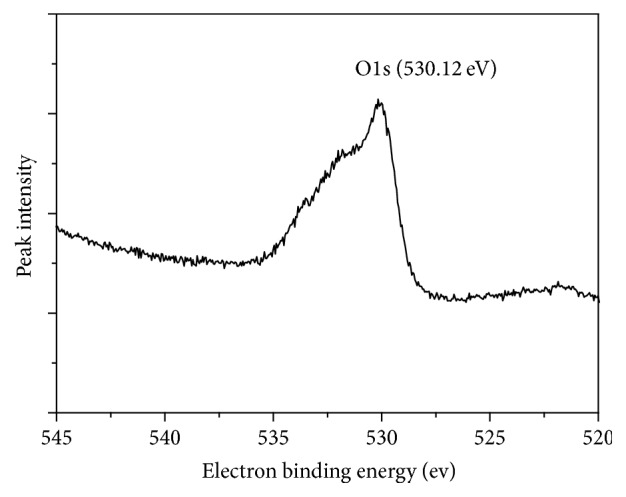
The spectrum of O1s before adsorption.

**Figure 14:**
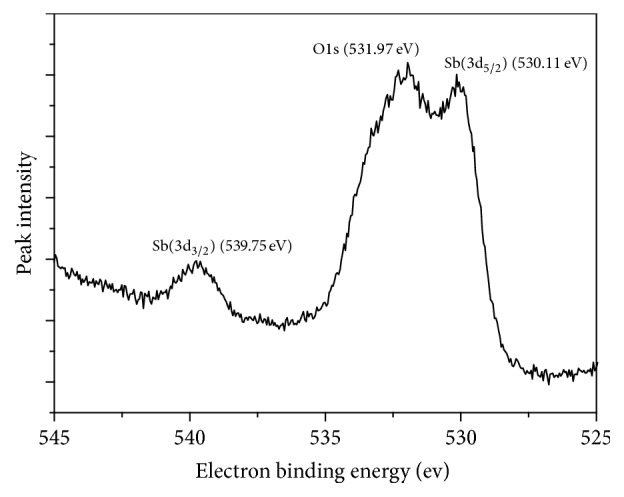
The spectrum of Sb3d and O1s after adsorption.

The XPS results of Fe_3_O_4_/GO after the adsorption of Sb(III) were depicted in Figure 12. There was a peak of Sb in the spectrum, which coincided with the peak of O1s. The spectrum of Sb after adsorption was presented in Figure 14. The spectra of the surface oxygen of Fe_3_O_4_/GO (before and after adsorption) were shown in Figures 13 and 14, respectively.

The binding energies of Sb(3d_5/2_) and Sb(3d_3/2_) detected by XPS were 530.11 eV and 539.75 eV. According to the XPS chemical state database, the superficial antimony on the Fe_3_O_4_/GO exists as Sb_2_O_5_, in which the antimony was pentavalent. However, the antimony of K(SbO)C_4_H_4_O_6_·1/2H_2_O used for preparing stock solutions was as a trivalent species. It demonstrated that the absorption process was accompanied with redox reaction, oxidizing the Sb III.

In order to further analyze the relation between bind energy displacement of O(1s) and surface charge, De Jong et al. [25] constructed a simplified mathematical model (10):

(10) Qo−4.372+385.023−8.976∗545.509−Xo1s1/2/4.488,

where *Q*_o_ is charge of oxygen atom (esu); *X*_o(1s)_ is binding energy measured by XPS (eV).

The charge variation of oxygen atom before and after the adsorption of Sb(III) onto GO was calculated by Formula (10), and the results are presented in Table 3.

Table 3 shows the charge of oxygen atom was changed from −0.871/esu to −0.738/esu during the adsorption of antimony onto Fe_3_O_4_/GO. Therefore, it could be concluded that the outer electron of oxygen atom transferred and the density of electrons changed during the adsorption process demonstrating that redox reaction occurred during the adsorption process.

### 3.6. Adsorbent Reuse

Reusability is a key factor in the investigation of adsorbent performance. A 0.1 mol/L of ethylene diamine tetraacetic acid (EDTA) solution was utilized in this experiment as a useful chelating agent to enhance removal of strongly adsorbed Sb and test reusability of the sorbent. The results showed Fe_3_O_4_/GO lost 50% of its efficiency after five continuous adsorption-regeneration-adsorption cycles as an adsorbent. This degradation may be due to decomposition of the sorbent or blocking of exchange sites by the EDTA.

## 4. Conclusions

The magnetic adsorbent Fe_3_O_4_/GO was synthesized and was studied in the removal of antimony from solution. The rate of removal rate of antimony was greatest in the pH range of 3.0–9.0. Reaction kinetics indicated that the adsorption of antimony onto Fe_3_O_4_/GO followed a pseudo-first-order kinetic model. The adsorption of Sb(III) onto synthesized Fe_3_O_4_/GO matched a Langmuir isothermal adsorption model according to reaction thermodynamics and the maximum adsorption capacity was found to be 9.59 mg/g. The thermodynamic parameters of the adsorption proved that the adsorption of Sb(III) onto Fe_3_O_4_/GO was a spontaneous and endothermic process. The XPS before and after adsorption and the relation between the binding energy displacement of O(1s) and surface charge indicated that the adsorption process was accompanied by a redox reaction. In addition, the regeneration test demonstrated that Fe_3_O_4_/GO composite has good reuse potential.

## Figures and Tables

**Table 1:** The adsorption isothermal eq. parameters of Fe_3_O_4_/GO.

*T* (K)	*q*_*e*,exp_ (mg/g)	Langmuir adsorption isotherm			Freundlich adsorption isotherm		
		*q*_max_ (mg/g)	*K*_*L*_ (L/mg)	*R* ^*2*^	*K*_*F*_	1/*n*	*R* ^*2*^
298	9.56	9.59	0.113	0.95	2.95	0.23	0.88

**Table 2:** The adsorption thermodynamic parameters of Sb(III) using Fe_3_O_4_/GO.

*T* (K)	Δ*G* (kJ/mol)	Δ*H* (kJ/mol)	Δ*S* (KJ/(mol·K))
298	−5.158		
308	−5.718	11.53	0.056
318	−6.278		

**Table 3:** Binding energy, charge change, and electron transfer direction of the oxygen atom in the adsorption process.

	Binding energy/eV	Electric charge/esu	Oxygen electron transfer direction
Fe_3_O_4_/GO	530.12	−0.871	
Fe_3_O_4_/GO + Sb	532.25	−0.738	Provide electronic

## References

[1] Sundar S., Chakravarty J. (2010). Antimony toxicity. *International Journal of Environmental Research and Public Health*.

[2] Yang X., Shi Z., Yuan M., Liu L. (2015). Adsorption of trivalent antimony from aqueous solution using graphene oxide: Kinetic and thermodynamic studies. *Journal of Chemical and Engineering Data*.

[3] Council of the European Communities. (1976). Council Directive 76/464/EEC of 4 may 1976 on pollution caused by certain dangerous substances discharged into the aquatic environment of the Community. *Official Journal L*.

[4] USEPA (1979). *Water related fate of the 129 priority pollutants*.

[5] Kang M., Kamei T., Magara Y. (2003). Comparing polyaluminum chloride and ferric chloride for antimony removal. *Water Research*.

[6] Leng Y., Guo W., Su S., Yi C., Xing L. (2012). Removal of antimony(III) from aqueous solution by graphene as an adsorbent. *Chemical Engineering Journal*.

[7] United States Environmental Protection Agency (1998). *Toxics Release Inventory*.

[8] Anderson C. G. (2012). The metallurgy of antimony. *Chemie der Erde - Geochemistry*.

[9] Wu Z., He M., Guo X., Zhou R. (2010). Removal of antimony (III) and antimony (V) from drinking water by ferric chloride coagulation: Competing ion effect and the mechanism analysis. *Separation and Purification Technology*.

[10] Gebel T. (1997). Aresnic and antimony: Comparative approach on mechanistic toxicology. *Chemico-Biological Interactions*.

[11] Oorts K., Smolders E., Degryse F. (2008). Solubility and toxicity of antimony trioxide (Sb2O3) in soil. *Environmental Science and Technology*.

[12] Filella M., Belzile N., Chen Y. (2002). Antimony in the environment: a review focused on natural waters: I. Occurence. *Earth-Science Reviews*.

[13] Zhao G., Li J., Ren X., Chen C., Wang X. (2011). Few-layered graphene oxide nanosheets as superior sorbents for heavy metal ion pollution management. *Environmental Science and Technology*.

[14] Jiao T. F., Liu Y. Z., Wu Y. T. (2015). Facile and scalable preparation of graphene oxide-based magnetic hybrids for fast and highly efficient removal of organic dyes. *Scientific Reports*.

[15] Teo P. S., Lim H. N., Huang N. M., Chia C. H., Harrison I. (2012). Room temperature *in situ* chemical synthesis of Fe_3_O_4_/graphene. *Ceramics International*.

[16] Chang Y.-P., Ren C.-L., Qu J.-C., Chen X.-G. (2012). Preparation and characterization of Fe_3_O_4_/graphene nanocomposite and investigation of its adsorption performance for aniline and *p*-chloroaniline. *Applied Surface Science*.

[17] Yang X., Shi Z., Liu L. (2015). Adsorption of Sb(III) from aqueous solution by QFGO particles in batch and fixed-bed systems. *Chemical Engineering Journal*.

[18] Zong P., Wang S., Zhao Y., Wang H., Pan H., He C. (2013). Synthesis and application of magnetic graphene/iron oxides composite for the removal of U(VI) from aqueous solutions. *Chemical Engineering Journal*.

[19] Hao Y., Wang Z., Gou J., Wang Z. (2015). Kinetics and thermodynamics of diquat removal from water using magnetic graphene oxide nanocomposite. *Canadian Journal of Chemical Engineering*.

[20] Kang M., Kawasaki M., Tamada S., Kamei T., Magara Y. (2000). Effect of pH on the removal of arsenic and antimony using reverse osmosis membranes. *Desalination*.

[21] Langmuir I. (1918). The adsorption of gases on plane surfaces of glass, mica and platinum. *The Journal of the American Chemical Society*.

[22] Freundlich H. M. F. (1906). Uber die adsorption in lusungen. *The Journal of Physical Chemistry*.

[23] Sari A., Çitak D., Tuzen M. (2010). Equilibrium, thermodynamic and kinetic studies on adsorption of Sb(III) from aqueous solution using low-cost natural diatomite. *Chemical Engineering Journal*.

[24] Xi J., He M., Lin C. (2011). Adsorption of antimony(III) and antimony(V) on bentonite: Kinetics, thermodynamics and anion competition. *Microchemical Journal*.

[25] De Jong B. H. W. S., Ellerbroek D., Spek A. L. (1994). Low‐temperature structure of lithium nesosilicate, Li_4_SiO_4_, and its Li1s and O1s X‐ray photoelectron spectrum. *Acta Crystallographica Section B*.

